# Overweight in adult cats: a cross-sectional study

**DOI:** 10.1186/s13028-018-0359-7

**Published:** 2018-01-19

**Authors:** Malin Öhlund, Malin Palmgren, Bodil Ström Holst

**Affiliations:** 1 Department of Clinical Sciences, Swedish University of Agricultural Sciences, P.O. Box 7054, 750 07 Uppsala, Sweden; 2 Kumla Animal Hospital, Företagsgatan 7, 692 71 Kumla, Sweden

**Keywords:** Dry food, Epidemiology, Feline, Logistic regression, Obesity, Type 2 diabetes

## Abstract

**Background:**

Overweight in cats is a major risk factor for diabetes mellitus and has also been associated with other disorders. Overweight and obesity are believed to be increasing problems in cats, as is currently seen in people, with important health consequences. The objectives of the present study were to determine the prevalence of overweight in cats from two different cohorts in a cross-sectional study design and to assess associations between overweight and diagnoses, and between overweight and demographic and environmental factors. Data were obtained from medical records for cats (n = 1072) visiting an academic medical center during 2013–2015, and from a questionnaire on insured cats (n = 1665). From the medical records, information on body condition score, breed, age, sex, neutering status, and diagnosis was obtained. The questionnaire included questions relating to the cat’s body condition, breed, age, sex, neutering status, outdoor access, activity level, and diet. Data were analyzed by multivariable logistic regression.

**Results:**

The prevalence of overweight was 45% in the medical records cohort and 22% in the questionnaire cohort, where owners judged their pet’s body condition. Overweight cats in the medical records cohort were more likely to be diagnosed with lower urinary tract disease, diabetes mellitus, respiratory disease, skin disorders, locomotor disease, and trauma. Eating predominantly dry food, being a greedy eater, and inactivity were factors associated with an increased risk of overweight in the final model in the questionnaire cohort. In both cohorts, the Birman and Persian breeds, and geriatric cats, were less likely to be overweight, and male cats were more likely to be overweight.

**Conclusions:**

The prevalence of overweight cats (45%) as assessed by trained personnel was high and in the same range as previously reported. Birman and Persian cats had a lower risk of overweight. The association with dry food found in adult, neutered cats is potentially important because this type of food is commonly fed to cats worldwide, and warrants further attention. Drawbacks related to the study design need to be acknowledged when interpreting the results, such as a potential for selection bias for cats visiting an animal hospital, and an information bias for questionnaire data. The high occurrence of overweight in cats needs to be addressed because it negatively affects their health.

**Electronic supplementary material:**

The online version of this article (10.1186/s13028-018-0359-7) contains supplementary material, which is available to authorized users.

## Background

Overweight and obesity are considered to be increasing problems in cats [1]. In humans, overweight and obesity are rapidly escalating problems, contributing to the global epidemic of type 2 diabetes mellitus (T2DM) [2, 3]. Overweight and obesity are also important risk factors for diabetes mellitus (DM) in cats [4–7]. The prevalence of overweight and obesity in cats ranges from 7 to 63% in different populations [8–16]. Obesity, together with physical inactivity, are believed to be the main contributors to the insulin resistance associated with T2DM in both cats and people [17–19]. Furthermore, overweight and obesity are associated with an increased risk of other diseases, such as lower urinary tract disease, dermatoses, oral cavity disease, and lameness [4, 6, 20]. Skin problems associated with overweight and obesity are mostly non-allergic, and authors speculate about whether an inability to groom is a contributing factor to this association [4]. An increased load on weight-bearing joints leading to osteoarthritis (OA) is one explanation for the increased risk of lameness in overweight cats, but human studies have also reported an increased risk of OA even in non-weight-bearing joints, suggesting a more general metabolic abnormality affecting the joint cartilage [4, 21]. In people, cats, and dogs, obesity is associated with an increased prevalence of certain types of cancer, and with a shortened life span [6, 22–25]. Obesity is in itself also considered a major animal welfare problem [26].

There are many studies on prevalence and risk factors for overweight and obesity in people, but studies on cats are scarcer. In Sweden, there are currently no studies reporting prevalence of overweight in cats. Better knowledge on predisposing factors for overweight and obesity is important to identify cats at risk at an earlier stage, to enable use of preventive measures to avoid overweight, and subsequently have a better possibility to prevent development of obesity-related diseases such as DM. The aims of this cross-sectional study were to determine the prevalence of overweight in adult cats, to assess associations between overweight and demographic factors and diagnoses in a cohort of cats visiting a University Animal Hospital, and to add the evaluation on associations between overweight and environmental factors derived from questionnaire data obtained from a cohort of adult, insured cats. Overweight in this study is defined as having a body condition score (BCS) above normal, which therefore includes both overweight and obese cats.

## Methods

### Study populations

#### Medical records cohort

This cohort consisted of medical records from all cats (n = 5935) visiting the University Animal Hospital, Swedish University of Agricultural Sciences, during 2013–2014, and records from only purebred cats during the first three quarters of 2015, to collect more pure-bred cat data to enable comparisons between breeds. Medical records were reviewed, and information on BCS, breed, age, sex, neutering status, and diagnosis was obtained. Cats were excluded if younger than 1 year of age at time of the visit to avoid cats that were not fully grown. If BCS was not assessed at all by the examining veterinarian or veterinary student, or if not assessed with a nine-grade scale (the BCS scale normally used at the hospital [27]), cats were also excluded from the study. Cats 1–2 years of age were grouped as “junior”, 3–6 years as “prime”, 7–10 years as “mature”, 11–14 years as “senior”, and 15 years of age or older as “geriatric”. Only one visit per cat was included in the dataset. Purebred cats with less than 20 individuals within the breed were grouped as “other purebreds”. Cats were grouped as “overweight” for BCS 6–9, which included both overweight and obese cats, and to “not overweight” for scores 1–5, which included normal weight and underweight cats [27, 28]. Diagnostic codes were assigned by the attending veterinarian based on a standardized system with about 8000 diagnostic codes available [29]. Diagnostic codes used were grouped into the following 12 categories: the whole animal (including unspecific diagnoses such as anorexia, fever, and depression), circulatory organs, digestive tract, DM, endocrine diseases other than DM, skin disorders, locomotor apparatus, respiratory tract, upper urinary tract, lower urinary tract, neoplasia, and traumatic injuries. The group with a diagnosis referring to the whole animal was used as a reference for comparisons because the group was large and had a normal mean BCS.

#### Questionnaire cohort

The second cohort comprised cats (n = 5363) insured by Agria Pet Insurance^1^ at any time during 2009–2013. The cats were used in a previous study as a non-diabetic control group, supplying data from a large cohort of randomly selected insured cats. Only cats 5 years of age or older were included in the previous study; therefore, data from younger cats were not available in the questionnaire cohort. Besides this no other selection was made for the cats included in this cohort [7]. All cat owners received an invitation to participate in the study by mail, including a web address to the survey. A web-based questionnaire containing 48 questions (in Swedish) was available during a 4-month period during 2015–2016 through an online survey provider.^2^ Questions included information about the age and sex of the respondent, number of adults in the household, the presence of children (< 18 years) in the household, the habitat, as well as questions on the cat’s birth year, breed, sex, and neutering status. Owners were asked if their cat was still alive, and if it was not, the cause and year of death. All questions on environmental factors referred to the last year of the cat’s life if the cat was no longer alive, or to the most recent year for cats alive at the time of the study. Owners were asked about the cat’s body condition, type of diet, feeding regimen, eating behavior, number of cats in the household, presence of other animals in the household, vaccinations, activity level, and about indoor confinement or whether the cat had access to the outdoors. Respondents were given several answering options per question, including the alternative “Other/I wish not to answer this question/I do not know”, as well as space for free-text answers. Answers were mandatory to proceed through the survey, and most often with only one answer per question possible. It was possible for respondents to return to previous questions and change their answers. Depending on the answer, some questions led the respondents to a set of extra questions. Questions on type of diet allowed several answers. Owners were asked to give only one answer if the cat’s diet consisted mainly of one type of food (≥ 75%), and two answers if the cat ate about 50% of each type of food. It was not possible to give more than two answers to this question. For the question on eating behavior, the respondent could choose between “greedy” (finishes meal immediately), finishes meal within hours, nibbles several times daily (grazer), or “picky” (leaves food). For activity level, the following options were available: very active, normal activity level, and inactive. Alternatives for the question on feeding regimen were ad libitum, once daily, twice daily, or three times daily.

All answers from the questionnaire were thoroughly scrutinized and all incomplete answers were excluded. In case of conflicting answers, e.g. birth year after time of death, contact with the respondent was made if possible, and answers were then corrected or excluded. All free text answers were reviewed and replies were corrected in case of obvious misinterpretation of the question, e.g. a change of breed category to “domestic” if the owner stated that the cat was in fact a mix between two breeds. Purebred cats with less than 20 individuals within the breed were grouped as “other purebreds”. Cats 5–6 years of age were grouped as “prime”, 7–10 years as “mature”, 11–14 years as “senior”, and 15 years or older as “geriatric”. Answers on type of diet were grouped according to the following categories: “dry” if ≥ 75% dry food, “mixed” if about 50% dry and 50% wet food, and “wet” if ≥ 75% wet food. Wet food included all types of wet or other high moisture food, excluding table scraps.

Respondents estimated their cat’s BCS using a five-grade scale with illustrations and accompanying instructions [30]. We chose the five-grade scale in order to facilitate body condition scoring for the owners. Cats were grouped to “overweight”, with estimated BCS of 4–5, which included both overweight and obese cats, and to “not overweight” for scores 1–3, which included normal weight and underweight cats.

### Data analysis

Data on BCS was dichotomized into the groups overweight and not overweight to be able to use the same type of regression analysis for both cohorts because different scales were used for the body condition scoring. The outcome of interest in the present study was overweight, defining both overweight and obese cats as overweight, which was the second reason for dichotomizing the outcome for analysis. Univariable logistic regression was used to assess associations between overweight and all explanatory variables included in the questionnaire cohort and the medical records cohort separately. Potential 2-way interactions were also assessed between all variables in each cohort. Multicollinearity between explanatory variables was not assessed. The answering option “I wish not to answer this question/I do not know” rendered very few replies per question, and these answers were therefore imputed to the most commonly used answering alternative if used by less than 2% of respondents. A backwards elimination approach with stepwise removal of nonsignificant main effects was then applied to a multivariable logistic regression analysis performed on each dataset, based on a lowered Akaike information criterion (AIC) as a measure of best goodness of fit of the final statistical model. Odds ratios (OR) for risk of overweight for all significant variables from the multivariable regression analysis were calculated in SAS by exponentiation of the parameter estimates, with 95% confidence intervals (CI). Comparisons between cohorts were performed with a *t*-*test* for mean age, and a Chi square test for the proportions of male, neutered, domestic, and overweight cats. Mean BCS and standard deviation (SD) was calculated for the different diagnostic code groups, and mean age and SD for each cohort. The significance level was set at 5%. Data handling was performed using SAS (version 9.4).^3^

## Results

### Medical records cohort

A total of 1157 medical records (19%) contained information on BCS and were included, while records from 4778 cats lacked information on BCS and were therefore excluded. An analysis of the medical records lacking information on BCS was not included in this study. Fourteen cats were excluded for using a scale other than the nine-grade. A total of 71 cats were then excluded for being younger than 1 year of age, leaving 1072 individual cats for analysis (18% of all cats visiting the hospital). Cats were predominantly domestic (65%). Fifty-seven percent were male and 43% female. Seventy-eight percent of cats were neutered according to the information available in the medical records. Mean age was 8.3 ± 4.6 years (range 1–21 years).

### Questionnaire cohort

The response rate was 32%, with a total of 1716 questionnaires received, of which 1686 were complete. A non-respondent analysis was not performed, due to confidentiality, because the respondents most often were anonymous. Twenty-one cats (1.2%) were excluded due to conflicting answers or because birth year was outside the selected range. Questionnaires with data on 1665 cats thus remained for analysis (31% of all cats). Cat owner characteristics did not differ between overweight and non-overweight cats. Respondents comprised 84% females and 16% males. About half of the respondents (47%) lived in towns (200–200,000 inhabitants), 26% lived in larger cities and 27% in the countryside. Seventy-eight percent of the cats lived in a household without children and 25% of the cats lived in a single-person household.

Most cats in the questionnaire cohort were domestic cats (80%), and 20% were purebreds, which was proportionally fewer than in the medical records cohort (P < 0.0001). Fifty-two percent were males and 48% were females, which also differed from the questionnaire cohort (P = 0.04). There were 0.2% intact males, and 1.7% intact females, leaving 98% of cats neutered, which was more than in the medical records cohort (P < 0.0001) Mean age was 13.9 ± 3.1 years (range 5–25 years), higher than for the medical records cohort (P < 0.0001). Fifty-nine percent of the cats were alive at the time their owners took part in the survey.

### Prevalence of overweight and results from the regression analyses

There were more overweight cats in the medical records cohort (45%) than in the questionnaire (22%) cohort (P < 0.0001). Descriptive statistics for cats in both cohorts are shown in Table 1, and diagnostic code groups including mean BCS for cats in the medical records cohort in Table 2.

**Table 1:** Descriptive statistics for both cohorts

	Medical records cohort (n = 1072)		Questionnaire cohort (n = 1665)	
	Total	Number of overweight cats (% within row)	Total	Number of overweight cats (% within row)
Breed (n)				
Birman	43 (4%)	4 (9%)	69 (4%)	2 (3%)
British shorthair	26 (2%)	17 (65%)	n.a.	n.a.
Cornish rex	20 (2%)	8 (40%)	n.a.	n.a.
Domestic cats	701 (65%)*	331 (47%)	1338 (80%)	317 (24%)
Maine Coon	50 (5%)	22 (44%)	20 (1%)	5 (25%)
Norwegian forest cat	51 (5%)	20 (39%)	61 (4%)	7 (11%)
Other purebreds	117 (11%)	58 (50%)	132 (8%)	32 (24%)
Persian	28 (3%)	5 (18%)	45 (3%)	6 (13%)
Ragdoll	36 (3%)	14 (39%)	n.a.	n.a.
Age (years)				
Mean (SD)	8.3 (± 4.6)*	n.a.	13.9 (± 3.1)	n.a.
Sex (n)				
Male	606 (57%)^†^	307 (51%)	868 (52%)	214 (25%)
Neutering status (n)				
Neutered	841 (78%)*	396 (47%)	1633 (98%)	364 (22%)
Body condition (n)				
Overweight	479 (45%)*	n.a.	369 (22%)	n.a.

General information on cats in the medical records cohort (n = 1072) and questionnaire cohort (n = 1665) with regard to breed, age, sex, neutering status, and body condition, including the number and proportion of overweight cats

*n.a.* not applicable, *SD* standard deviation

* Difference between cohorts P < 0.0001

^†^Difference between cohorts P = 0.04

**Table 2:** Diagnostic code groups and body condition

Diagnostic code group	Number of cats (n = 1072)	Mean body condition score (± SD)
Lower urinary tract	89 (8.3%)	6.2 (± 1.6)
Locomotor apparatus	53 (4.9%)	6.0 (± 1.6)
Respiratory tract	53 (4.9%)	5.9 (± 1.7)
Skin disorders	66 (6.2%)	5.9 (± 1.5)
Diabetes mellitus	23 (2%)	5.8 (± 2.0)
Trauma	160 (14.9%)	5.6 (± 1.3)
Neoplasia	47 (4.4%)	5.2 (± 1.8)
Digestive tract	165 (15.4%)	5.2 (± 1.7)
The whole animal	273 (25.5%)	4.8 (± 1.9)
Circulatory system	40 (3.7%)	4.7 (± 1.7)
Endocrine (other)	32 (3.0%)	4.4 (± 1.5)
Upper urinary system	71 (6.6%)	4.3 (± 1.7)

Number of cats in the medical records cohort (n = 1072) per diagnostic code group, and mean body condition score (scale 1–9) in each group

*SD* standard deviation

**Fig. 1:**
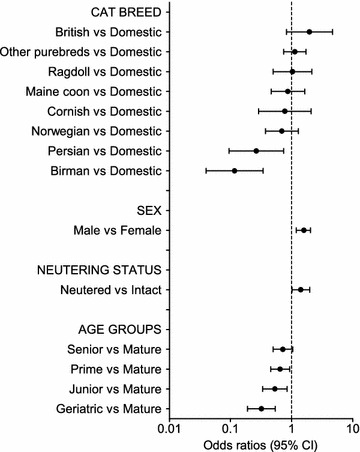
Multivariable logistic regression on the medical records cohort—demographic factors. Odds ratios for overweight in the medical records cohort (overweight cats n = 479, not overweight n = 593) from the multivariable logistic regression analysis depending on demographic risk factors (breed, sex, neutering status, and age groups). Error bars represent 95% confidence intervals (CI)

**Fig. 2:**
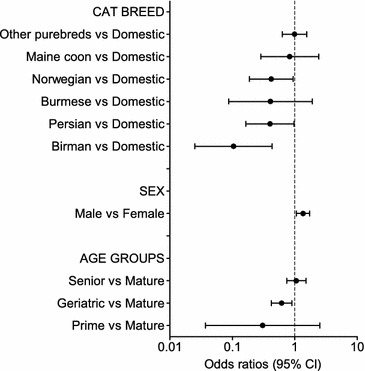
Multivariable logistic regression on the questionnaire cohort—demographic factors. Odds ratios for overweight in the questionnaire cohort (overweight cats n = 369, not overweight n = 1296) from the multivariable logistic regression analysis depending on demographic risk factors (breed, sex, and age groups). Error bars represent 95% confidence intervals (CI)

**Fig. 3:**
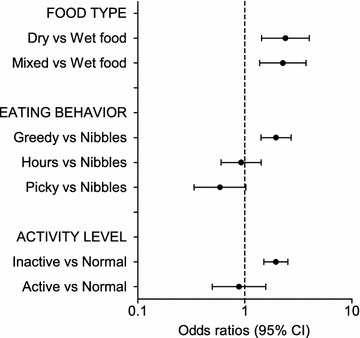
Multivariable logistic regression on the questionnaire cohort—environmental factors. Odds ratios for overweight in the questionnaire cohort (overweight cats n = 369, not overweight n = 1296) from the multivariable logistic regression analysis depending on environmental risk factors (food type, eating behavior, and activity level). Error bars represent 95% confidence intervals (CI)

**Fig. 4:**
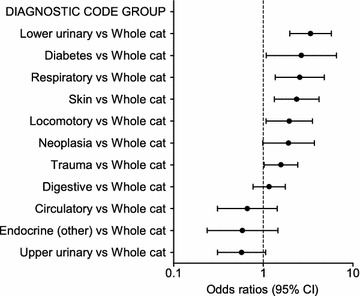
Multivariable logistic regression on the medical records cohort—diagnostic code groups. Odds ratios for overweight in the medical records cohort (overweight cats n = 479, not overweight n = 593) from the multivariable logistic regression analysis depending on diagnostic code group. Error bars represent 95% confidence intervals (CI)

In the univariable analysis on data from the medical records cohort, the following variables were associated with overweight: breed (P = 0.0002), sex (P < 0.0001), age (P < 0.0001), neutering status (P = 0.0026), and diagnostic code group (P < 0.0001). In the questionnaire cohort, the following variables were associated with overweight: breed (P = 0.0038), sex (P = 0.015), age (P = 0.0007), type of food (P = 0.0031), eating behavior (P < 0.0001), and activity level (P < 0.0001). There were no significant interactions present between any of the variables. All variables showing significance in the univariable analyses remained significant in the multivariable logistic regression for both cohorts. Results from the multivariable logistic regression analyses are shown in Figs. 1, 2, 3 and 4. Odds ratios including CIs, and P values, for all significant variables are shown in Additional file 1.

In both cohorts, Birman and Persian cats had a lower risk of overweight than domestic cats. In the questionnaire cohort, the Norwegian forest cat breed also showed a decreased risk of overweight. Domestic cats had an increased risk of overweight compared with purebreds in the questionnaire cohort (OR 1.8; 95% CI 1.3–2.5).

Male cats were at increased risk of overweight compared with females in both cohorts (OR 1.6; 95% CI 1.2–2.0 in the medical records cohort, and OR 1.4; 95% CI 1.1–1.7 in the questionnaire cohort). Neutering status was only significant in the medical records cohort, where neutering was associated with an increased risk of being overweight (OR 1.4; 95% CI 1.0–2.0).

In both cohorts, geriatric cats were less likely to be overweight than mature cats (OR 0.3; 95% CI 0.2–0.5 and OR 0.6; 95% CI 0.4–0.9). In the medical records cohort, junior and prime cats were also less likely to be overweight (OR 0.5; 95% CI 0.3–0.8 and OR 0.7; 95% CI 0.5–0.9), compared with mature cats.

In the medical records cohort, several diagnostic code groups were associated with overweight when compared with cats with a diagnosis referring to the whole animal. Cats were more often overweight if they had a diagnosis related to the lower urinary tract (OR 3.4; 95% CI 2.0–5.7), DM (OR 2.7; 95% CI 1.1–6.6), respiratory tract (OR 2.6; 95% CI 1.4–4.8), skin (OR 2.4; 95% CI 1.3–4.2), the locomotor system (OR 1.9; 95% CI 1.1–3.5), or related to trauma (OR 1.6; 95% CI 1.0–2.4).

In the questionnaire cohort, eating predominantly dry food was associated with an increased risk of overweight compared with wet food (OR 2.4; 95% CI 1.4–4.0). Being defined as a greedy eater was also associated with overweight (OR 2.0; 95% CI 1.4–2.7) as compared with cats that preferably nibble several times daily (grazer). Inactive cats were more likely to be overweight compared with cats with a normal activity level (OR 2.0; 95% CI 1.5–2.5).

Variables not associated with overweight in the questionnaire cohort were cat owner characteristics (number of adults in the household, P = 0.79; presence of children in the household, P = 0.93; owner sex, P = 0.21; owner age, P = 0.71), the habitat (P = 0.17), feeding regime (P = 0.22), vaccination status (P = 0.07), number of cats in the household (P = 0.33), presence of other animals in the household (P = 0.67), and outdoor access or indoor confinement (P = 0.43).

## Discussion

The prevalence of overweight in adult cats visiting an academic medical center in our study was high, with almost every second cat considered overweight when body condition was assessed by a veterinarian or veterinary student. In the second cohort, where owners estimated their pets’ body condition, the prevalence of overweight was lower, with one in five cats considered overweight. The prevalence of overweight found in this study was in the same range as in previous reports [8–11]. Different scales were used for scoring, a nine-grade for the medical records cohort and a five-grade scale for the questionnaire cohort, but because all cats were grouped into only two groups for the analyses, overweight versus not overweight, the influence of using different scales would in our opinion be slight. The divergence between the cohorts can be explained by the actual differences present between the cohorts. There were differences in breed composition, mean age, number of male versus female cats, and neutering status present between cohorts, but the larger proportion of domestic, neutered and older cats in the questionnaire cohort would implicate a higher prevalence of overweight in this group, which was in contrast to what was found. It has previously been described that there is a tendency of owners to underestimate the BCS of their pet, which probably contributes to the lower prevalence seen in the questionnaire cohort [12, 31, 32]. Courcier et al. [31] found that owner misperception was more likely when owners rated cats with BCS 1 (very thin) and 4 (overweight) on a five-grade scale, and in longhaired cats. Moreover, it has been shown that owner underestimation of the cat’s BCS is in itself a risk factor for obesity [10, 12, 32]. Because owners tend to underestimate their pet’s BCS, we believe that cats judged as overweight in the questionnaire are in fact truly so, strengthening the associations found with overweight in the study. All cats visiting the University Animal Hospital are supposed to have an assessment of their BCS, but in reality only one in five cats was scored, and there is probably also a selection of which cats are actually selected for scoring, which are limitations of the study. It is unknown whether there is a tendency to more often score obese cats, or thin cats. It is possible that healthy cats undergoing routine prophylactic procedures such as vaccinations, and critically ill cats, are not being scored to the same extent as other cats. How this affects our results is unknown. Moreover, cats visiting an animal hospital might not be representative of the general population.

Several diagnostic code groups were associated with overweight. Scarlett and Donoghue reported associations between obesity and lameness, DM, and non-allergic skin disorders [4], similar to the findings in our study, and Lund et al. found associations between obesity and urinary tract disorders [6], also found in our study. Excess weight affects joints mechanically and can lead to OA, but it has also been shown in people that arthrosis is a hormonally mediated disease associated with obesity [21]. The association between DM and overweight in cats is well-known and supported by our findings [1, 4, 7]. Scarlett and Donoghue found a fourfold increased risk of DM in obese cats [4]. Lower urinary tract disease such as urethral obstruction is commonly seen in overweight, neutered, middle-aged male cats [33], similar to our findings. It is not clear from our cross-sectional study design if overweight predisposes cats to certain diseases, or if being overweight is a consequence of disease. It is possible that if overweight predisposes cats to disease, more overweight cats will be encountered at an animal hospital than in the general population.

We found that cats eating predominantly dry food were more often overweight than cats eating predominantly wet food, in a cohort consisting of mature and mainly neutered cats. An association between dry food and overweight in adult cats has to our knowledge not been reported before, although recent studies on younger cats showed feeding a dry diet to be a risk factor for overweight [14, 15]. Rowe et al. [14] showed that cats fed dry food as the only or major part of their diet at about 2 years of age, were twice as likely to be obese compared to those fed a wet or a mixed diet. Because dry food is fed to a vast number of cats worldwide, this finding warrants further investigation. Our group has previously shown an association between dry food and an increased risk of DM in cats assessed as normal weight by their owners [7]. Many studies have investigated associations between food type and risk of overweight, but only associations with therapeutic and premium dry diets have been found, a finding likely to be a confounder because weight loss diets are often prescribed to overweight patients [6, 9, 10, 31, 34–36]. However, the change from a low-carbohydrate diet in feral cats consuming wild prey to a typical high-carbohydrate diet fed to many cats today, has been suggested to be partially responsible for the recent increase in obesity and DM seen in domestic cats [17]. In 1963, Joshua [37] stated that a diet change was not necessary for cats diagnosed with DM because they were already on a very low carbohydrate intake anyway, which reflects the difference in how cats were fed then compared to now, with many cats fed a high-carbohydrate diet such as dry food. High-carbohydrate diets have been shown to lead to higher insulin blood concentrations than high-protein and high-fat diets [38]. The exact macronutrient content in food given to cats in our study is unknown, although a typical commercial dry diet generally contains more carbohydrates than a typical wet diet [39]. Dry food is typically more energy-dense than wet food, which contains more water, which also may contribute to our findings [40]. Cats have been shown to decrease their voluntary energy intake when fed a canned diet *ad lib* compared with a freeze-dried version of the canned diet, indicating that the bulk water might promote weight loss in cats [41]. Because it is not possible to alter one macronutrient without altering another, the difference in BCS between cats fed dry food and cats fed wet food might relate to a protein effect rather than a carbohydrate effect. Studies have shown that increased dietary protein promotes fat loss and reduces loss of lean body mass during weight loss in cats [42]. It has also been shown that high-protein diets can increase energy expenditure, as protein can induce a higher thermic effect than the other macronutrients [43]. Cats are obligate carnivores, whose natural diet consists mainly of protein-rich animal prey [44]. Moreover, cats lack several enzymes involved in carbohydrate metabolism, such as salivary amylase, and have low activities of intestinal amylase and disaccharidases, indicating that they are not adapted to using carbohydrates as a primary energy source, although they can still digest and utilize cooked starch [45, 46].

There was no association between feeding regimen and overweight in our study, in contrast to some previous studies showing conflicting results, with both ad libitum feeding and being fed twice daily as risk factors for obesity [31, 36]. Being defined as a greedy eater, however, was associated with an increased risk of being overweight. Being greedy was an independent risk factor also for DM in a previous study from our group [7]. In people, eating slowly is associated with a lower caloric intake and enhanced satiety [47], but this has to our knowledge not been evaluated in cats.

Activity level was associated with overweight in our study, with inactive cats at higher risk. On the other hand, we could not detect an association between indoor confinement and access to the outdoors and overweight. Some studies have shown indoor confinement to be a risk factor for obesity [9, 15, 34], whereas others have failed to show such an association [12, 31, 36]. Inactivity has been reported as a risk factor for obesity, but others have reported no associations between activity and obesity [9, 10]. According to the present study, it is the activity in itself that is important, not whether it is performed outdoors or indoors. However, in a previous study from our group, investigating risk factors for DM in cats, the opposite was found, with indoor confinement being a more important risk factor for disease than the activity levels [7]. It should be noted that the measurement of activity level is subjectively made, and performed by the owners. Future studies investigating the effect of activity on body weight will benefit from using objective measurements of the cats’ activity levels.

Male sex was associated with an increased risk of overweight in both cohorts, similar to what has previously been shown [6, 11, 12, 18, 48]. Male cats have been shown to gain weight more easily than female cats [18]. Neutering was a risk factor found only in the medical records cohort, because almost all cats in the questionnaire cohort were neutered, making comparisons between neutering statuses unfeasible. Neutering can increase daily food intake, decrease the metabolic rate, and cause activity levels to drop, thereby predisposing neutered cats to obesity [48–52]. Caloric restriction is generally required after neutering, and a failure to adjust food supply to meet the lower energy requirements can easily lead to obesity in the neutered cat [50]. Both male sex and neutering have been identified as risk factors also for DM [5, 53]. It is not clear whether the neutering itself causes insulin resistance or whether it indirectly influences the risk of DM by increasing the risk of obesity.

Geriatric cats were less likely to be overweight compared with mature cats in both cohorts, in concordance with previous studies [6, 11, 13, 34, 36]. Sarcopenia is a natural age-related change that likely contributes to this finding, as well as the presence of concurrent diseases causing weight loss more commonly seen in older cats, such as chronic kidney disease, hyperthyroidism, and dental problems [54]. In the medical records cohort, the junior and prime age groups showed less overweight compared with mature cats, but in the questionnaire cohort, the lack of cats younger than 5 years excluded comparisons. It is interesting that the age incidence of DM in cats closely follows the age incidence of overweight, again stressing the importance of obesity as a major risk factor for DM [53].

Birman and Persian cat breeds showed a decreased risk of overweight in our study. We did not identify any particular cat breed at an increased risk of being overweight, but when comparing purebred and domestic cats in the questionnaire cohort, the domestic cats were more often overweight, similar to what has been shown previously [6, 12, 13]. There is also an increased risk for DM in domestic cats compared with purebred cats [53]. The association between breed and overweight likely to some extent reflects the genetic aspect of the condition. Haring et al. have recently shown that a genetic component is responsible for the development of overweight in cats [55]. In the questionnaire cohort, with owners estimating their pet’s body condition, the Norwegian cat breed showed a decreased risk of obesity, differing from previous studies [56]. It also differed from our expectations, because the Norwegian forest cat has been shown to have a breed predisposition to DM, and a propensity for obesity was anticipated [53]. The Norwegian forest cat is according to the breed standard a large cat breed, which might lead owners to underestimate their pet’s body condition. It should be noted that in the medical record cohort, when BCS was assessed by a veterinarian or veterinary student, the Norwegian forest cats did not differ in body condition compared with other pedigree cats.

Limitations of our study are mainly related to the study design, particularly the problems with recall bias, because some of the answers in the questionnaire referred to several years back in time, and also the difficulties for owners to accurately assess their cats’ BCS which can be a significant confounder. The multiple assessors of the BCS is also a drawback of the study. Moreover, the potential selection bias may also affect the estimated associations of overweight with risk factors and diseases. On the other hand, associations between overweight and demographic factors including age, breed and sex, were similar between cohorts, strengthening the results.

## Conclusions

We found a high prevalence of overweight in adult cats, similar to other reports. Having a greedy eating behavior and being inactive were factors associated with overweight, as were diagnoses such as lower urinary tract disease, DM, respiratory disease, skin disorders and locomotor disease. In both cohorts, Birman and Persian cats, and geriatric cats, were less likely to be overweight, whereas male cats were more likely to be overweight. There was a divergence in the estimated occurrence of overweight between the cohorts, due to differences between groups, but probably also explained by the owners’ inability to correctly assess their pets’ body condition. Because overweight is a growing problem in both pets and people, there is a risk that our perception of what is actually a normal body condition is being slowly altered. Finally, the association found between dry food and overweight in the group of adult, neutered cats, warrants further investigation because dry food is a common food type fed cats worldwide.

## Additional file

**Additional file 1.** Results from the final models from the logistic regression analyses from the medical records cohort (n = 1072) and the questionnaire cohort (n = 1665). Odds ratios (OR) including 95% confidence intervals (CI), and P values, are shown for all significant variables.

## Abbreviations

BCS: body condition score
CI: confidence interval
DM: diabetes mellitus
OA: osteoarthritis
OR: odds ratio
SD: standard deviation
T2DM: type 2 diabetes mellitus

## Footnotes

1 Agria Pet Insurance, P.O. Box 70306, SE-107 23, Stockholm, Sweden.

2 Netigate®, Lästmakargatan 20, SE-111 44, Stockholm. Sweden.

3 SAS Institute Inc., Cary, NC 27513, US.
