# Supplementary material for: Overweight in adult cats: a cross-sectional study
Source: Acta Vet Scand. 2018 Jan 19;60:5. doi: 10.1186/s13028-018-0359-7 (PMC5775588; doi:10.1186/s13028-018-0359-7)
Supplement: Supplementary file 1 — Additional file 1. Results from the final models from the logistic regression analyses from the medical records cohort (n = 1072) and the questionnaire cohort (n = 1665). Odds ratios (OR) including 95% confidence intervals (CI), and P values, are shown for all significant variables. [file 13028_2018_359_MOESM1_ESM.pdf]

Additional file 1. Results from the final models from the logistic regression analyses from the medical records cohort (n = 1072) and the questionnaire cohort (n = 1665). Odds ratios (OR) including 95% confidence intervals (CI), and P values, are shown for all significant variables.

**Variables**	**Medical records cohort (n = 1072)**		**Questionnaire cohort (n = 1665)**	
	**OR (95% CI)**	***P* value**	**OR (95% CI)**	**P value**
**Breed**		0.0009		0.0033
Birman versus Domestic	0.12 (0.04–0.34)		0.11 (0.03-0.43)	
British versus Domestic	1.96 (0.83–4.66)		n.a.	
Cornish rex versus Domestic	0.77 (0.29–2.08)		n.a.	
Maine coon versus Domestic	0.87 (0.46–1.64)		0.83 (0.29-2.41)	
Norwegian versus Domestic	0.69 (0.37–1.29)		0.42 (0.19-0.94)	
Other breeds vs Domestic	1.13 (0.75– 1.73)		0.91 (0.59-1.41)	
Persian versus Domestic	0.27 (0.10–0.74)		0.40 (0.17-0.97)	
Ragdoll versus Domestic	1.04 (0.50–2.15)		n.a.	
**Sex**		0.0011		0.015
Male versus Female	1.56 (1.20–2.03)		1.35 (1.06-1.73)	
**Neutering**		0.040		n.s.
Neutered versus Intact	1.42 (1.02–1.97)		n.s.	
**Age group**		0.0003		0.0007
Junior versus Mature	0.53 (0.34–0.85)		n.a.	
Prime versus Mature	0.65 (0.46– 0.93)		0.31 (0.04-2.50)	
Senior versus Mature	0.72 (0.50–1.04)		1.05 (0.73-1.49)	
Geriatric versus Mature	0.32 (0.19–0.54)		0.61 (0.41-0.90)	
**Diagnosis/organs affected***		< 0.0001		n.a.
Lower urinary tract	3.37 (1.98–5.74)		n.a.	
Diabetes mellitus	2.66 (1.08–6.57)		n.a.	
Respiratory tract	2.55 (1.36–4.81)		n.a.	
Skin	2.36 (1.33–4.19)		n.a.	
Locomotor apparatus	1.95 (1.07–3.53)		n.a.	
Neoplasia	1.92 (0.99–3.71)		n.a.	
Trauma	1.57 (1.02–2.42)		n.a.	
Digestive tract	1.17 (0.77–1.76)		n.a.	
Circulatory system	0.66 (0.31–1.43)		n.a.	
Endocrine disease (excl. DM)	0.59 (0.24–1.46)		n.a.	
Upper urinary system	0.57 (0.31–1.07)		n.a.	
**Type of diet**		n.a.		0.0031
Dry versus Wet	n.a.		2.40 (1.44-4.00)	
Mixed versus Wet	n.a.		2.26 (1.37-3.72)	
**Activity**				< 0.0001
Inactive versus Normal	n.a.		1.94 (1.50-2.52)	
Active versus Normal	n.a.		0.89 (0.50-1.58)	
**Eating behavior**				< 0.0001
Greedy versus Nibbles	n.a.		1.94 (1.40-2.68)	
Finishes in hours versus Nibbles	n.a.		0.92 (0.60-1.42)	
Picky versus Nibbles	n.a.		0.59 (0.34-1.03)	

OR, odds ratio; CI, confidence interval; n.a., not applicable; n.s., not significant; DM, diabetes mellitus. * Diagnostic code group versus a diagnosis referring to the whole animal.
